# Subperitoneal Fibroid Tumor of the Uterus Removed through an Incision in the Posterior Wall of the Vagina

**Published:** 1876-12

**Authors:** R. Stansbury Sutton

**Affiliations:** Alleghany, Pa.


### SUBPERITONEAL FIBROID TUMOR OF THE UTERUS, REMOVED THROUGH AN INCISION IN THE POSTERIOR WALL OF THE VAGINA.

By R. STANSBURY SUTTON, A.M., M.D., of Alleghany, Pa.

Until February, 1870,[1] the history of surgery furnishes no instance in which a tumor growing within the peritoneal cavity was removed through the posterior wall of the vagina. The honor of having first suggested and performed the operation belongs to Dr. T. Gaillard Thomas, Professor of Obstetrics and Diseases of Women, in the College of Physicians and Surgeons, of New York. This case was one of Ovarian Tumor "the size of a large orange." The patient recovered.

In 1872,[2] Dr. R. Davis, of Wilkesbarre, Pa., removed by this route, an Ovarian Tumor, weighing 9 lbs. This patient recovered.

[1] Amer. Jour. Med. Sciences, April, 1870; also, Peaslee on Ovarian Tumors, p. 319.

[2] Trans. State Med. Soc. of Pa., 1874; also, Half Yearly Compend Med. Science, Jan. 1876.

[3] New Orleans Med. and Surg. Journal, Nov. 1873.

[4] Amer. Jour. Med. Sciences, July, 1876.

In 1873,[3] Dr. S. T. Gilmore, of Mobile, Alabama, had a successful case.

In 1874,[4] Dr. Robert Battey, of Atlanta, Ga., had a successful case.

Until the present year, four American Surgeons had successfully removed Ovarian Tumors *per vaginam*, and in no instance had the operation been followed by symptoms unusually severe. I have failed to find any record of the removal of subperitoneal fibroids by this method ; and so far as my knowledge extends, I do not know that the operation has ever been attempted, excepting by myself in the following case. I take the case from my note book.

June 19, 1876. Emma G——, a black woman, was admitted to my ward to-day, after my visit had been made.

June 20. She is fifty years of age, her skin is hot and dry, temperature 103°, tongue furred, edges red, has considerable tenderness over the abdomen, especially in the right iliac region. Her bowels have not been moved for more than a week, and she complains of a pain down the entire length of the right sciatic nerve and its terminal branches. Says she has a tumor in the pelvis. Ordered a sponge bath of tepid water, to be repeated occasionally, and a mixture of solution of citrate of magnesia, fluid extract of senna, and syrup of ginger, to be given immediately.

June 21. Temperature normal, tongue cleaning, the bowels have been moved freely, less abdominal tenderness exists.

An examination *per vagniam* reveals a large, solid tumor, ovoidal in shape, filling the right half of the pelvic cavity, and pushing the uterus firmly to the left lateral wall. The pressure of the finger against the tumor elicits pain, and the surface of the tumor is rough and as hard as bone. The sound enters the uterus just two and one-half inches. The uterus can be moved but the tumor cannot. With the sound in the right hand and the index finger of the left pushed hard in between the uterus and tumor, a connecting band betwen the uterus and tumor is discernable, beginning just above the vaginal attachment. The tumor pushes the posterior and right lateral vaginal walls downward and forward. A finger in the rectum finds the growth pressing upon the canal, and pressure elicits pain but no motion in the growth. She was now placed upon her knees with the chest resting upon the bed. A prolonged and severe effort by pressure with two fingers in the vagina, failed to more than barely move the tumor, and it was impossible to dislodge it from the pelvic cavity. Pressure with the left hand above Poupart's ligament in the right iliac region detects the growth.

*Diagnosis.* A subperitoneal fibroid tumor of the uterus, which is pediculated, and which has undergone cretaceous degeneration; which process has produced these inflammations, resulting in adhesions short and strong. This diagnosis was strengthened by the fact that she gave evidence of having had several attacks of fever, attended with severe pain in the lower part of the abdomen. These attacks in nearly every instance followed the effort necessary to do a day's washing. She sustained herself as a domestic,. but latterly found all exercise painful. Ordered 2½ grs. quiniæ sulphat. and ℥j elix. bismuthi after each meal. Good diet, sponge bath daily.

June 23. Ordered sol. magnesiæ citrat. with ext. sennæ. Operated satisfactorily.

June 24. I called the entire staff together to-day for consultation. By invitation, Dr. A. M. Pollock was present with us. The patient is feeling moderately comfortable, has no fever, tongue clear, skin moist, pulse rapid, nervous.

The diagnosis at which I had arrived was stated, and the patient being now under the influence of ether, Dr. Pollock was invited to examine the case. He introduced his hand into rectum and with considerable effort was able to get some movement in the growth, but found it impossible to dislodge it from the pelvis, after pursuing his examination to the end, the various members of the staff examined the case as carefully as possible without again introducing the hand into rectum. The ether was withdrawn, the patient left with the nurse, and we retired for consultation.

No opinion was offered by any of the staff — Dr. Pollock expressed himself as not entirely satisfied that the growth was a fibroid or connected with the uterus, that it was hard enough to be an exostosis. In reply to his question as to whether I was entirely satisfied, I replied that I was. We then passed on to the question of treatment. It was the opinion of all that medical treatment was in this case of no avail, and that an effort to remove it by an operation was of questionable propriety. To abandon the woman to her condition was to leave her to constant suffering and to the charity of the public for sustenance. She rebelled against this, preferring to die from an operation, rather than be abandoned thus. In view of all the facts, we decided to make an effort to remove the tumor, and as the operation devolved upon me, I was left to select the method.

June 25th, 11:30 A. M. After the examination yesterday, a full dose of opium was administered, and at 9 A. M. this morning 25 gtts. tinct. opii, were given by the mouth. The patient slept well last night and is in very fair condition this morning. The patient was placed upon the table on her left side in Sim's position, the bladder emptied with a catheter. Drs. McCoy and C. C. Lange took charge of the anæsthetic (Squibb's ether). Sims' speculum was now introduced and held by Dr. Pollock, while the limbs were guarded by the left hand and the right side of the nates elevated by the right hand of Dr. Purviance, who stood opposite to Dr. Pollock. In addition to the gentlemen mentioned there were present Drs. Estep and Gieseking of this city, and Dr. Hugh L. Guthrie of Sparta, Illinois. All being ready I proceeded to operate as follows:

The posterior wall of the vagina was seized about midway between the rectum and cervix uteri, with a tenaculum and cut through with one stroke of the scissors; with a probe pointed bistoury and the aid of a tenaculum, this incision was extended as far as possible toward the rectum and toward the cervix. All bleeding being arrested by sponging with cold water, the peritoneum was picked up with Sim's small tenaculum and cut through with the scissors. This incision was now made of the same length as the former, with the probe pointed bistoury. The finger was now readily brought in contact with the tumor. An effort made to enucleate the growth in its position failed. The hand, first dipped in carbolized water, was carried into the cavity of the pelvis, the tumor grasped and all its adhesions forcibly broken up. It was found to be attached by a fleshy pedicle to the posterior wall of the uterus. When the hand was withdrawn, the small intestines followed into the vagina. These were carefully pushed back with the hand, which was again carried up to the growth. A pair of strong vulsellum forceps were carried closed along the front of the wrist and palm of the hand and carefully expanded over the tumor, which was now seized and drawn into the vagina, the index finger of the left hand working back over the growth as much as possible the lips of the vaginal wound. The speculum was again introduced, and the exposed capsule incised as far as it could be reached and with the aid of a pair of dressing forceps, a tenaculum handle, and the finger nail, it was stripped back beyond the equator of the growth on all sides. The tumor now occupied the vagina and a second pair of forceps being fastened upon its stripped surface, the first pair were carefully removed. A pair of guarded hooks were also fastened into it and by these with Dr. Pollock's assistance, we pulled the tumor through the external outlet of the pelvis. Dr. Guthrie supporting the perineum with one hand and stripping back the capsule with the thumb nail of the other, as the tumor came out. Two vessels in the pedicle required ligation. The pedicle with some folds of small intestine in the vagina were now pushed back into the abdominal cavity. No stitches were applied in the vaginal wound. The patient had been forty minutes under the anæsthetic of which eight fluid ounces had been consumed.

The patient was now put to bed, pulse one hundred and twenty. Ordered $\frac{1}{8}$ gr. morphiæ sulph. every three hours, oftener if required.

4 P. M. Catheter used — to be used every six hours.

7:45 P. M. Pulse one hundred and thirty, skin hot, no pain. Continue $\frac{1}{8}$ gr. doses of morphia as required, and give five drops of tincture of verat. virid. every three hours. To have milk *ad libitum.*

June 26th, 9 A. M. Patient has slept some, pulse one hundred and eighteen, temperature 102°, slight nausea. Discontinue veratrum, give ten grs. quinine every three hours, continue morphia. The tubes of a stomach pump was carefully passed just beyond the vagina, and a quart of slightly carbolized warm water discharged through it, this returned at first cloudy then clear.

3 P. M. Pulse one hundred and fifty four, temperature $104\frac{9}{10}°$, great tympany, complains of pain.

5:45 P. M. Pulse one hundred and forty-eight, temperature 104°, tongue coated and dry. Repeated the douche. Pulse fell four beats, and temperature $\frac{1}{10}$ of one degree — continue quinine and morphia. To have ten drops of veratrum.

9 P. M. Pulse one hundred and thirty-six, temperature 101°. Pulse easily compressed, vomited at 7 P. M.

9:45. Passing gas off by the bowel.

June 27th, 9:15 A. M. Pulse one hundred and twelve, temperature $98\frac{1}{2}°$, tongue moist. Used the douche again and let the gas off the lower bowel with a tube passed into the rectum.

10:45 A. M. Patient sleeping, legs stretched out, seems comfortable, pulse one hundred and eight.

June 28th, 10 A. M. Has been restless for several hours, pulse one hundred and twenty, temperature 101°, complains of pain, has had no morphia all night. Had some discharge from bowels, morphia to be resumed.

12 M. Temperature 103°, pulse one hundred and thirty.

3 P. M. Temperature $104\frac{1}{5}°$, pulse one hundred and forty, tympany great, cerebral excitement.

9 P. M. Temperature not taken, pulse one hundred and sixty.

June 29th, 9 A. M. Patient died this morning.

*Post Mortem.* Present Drs. Sutton, Pollock and C. C. Lange.

A careful investigation revealed a wound of the small intestine, which had been made with a prong of the vulsellum during the operation. This wound was only discovered by making pressure along the intestines and finding that gas escaped at what was a mere pin hole, leading down from which was the line of a little rent which was so completely glued as to require some effort to separate the edges. At what stage in the operation this occurred I do not know. The danger was appreciated and careful attention given to avoid it. Doubtless the escape of gas was one cause of the peritonitis which carried her off.

No comment, probably, is necessary on this case, but I cannot resist giving expression to the impression it has left upon my mind. *First*, that fibroid or solid tumors are extremely difficult to remove by this method, and *second*, that all operations for the removal of tumors from the cavity of the peritoneum are more easily and safely performed through the linea alba.

---

### A CASE OF CLONIC CONVULSIONS.

Reported by Dr. C. A. ROOD, Monroe, Wis.

I was summoned in haste at 3 A. M., Monday, May 8th, to see Mr. H. E., aged twenty-one years, of a nervous temperament, suffering from convulsions. Upon arriving, found the young man sitting in an arm chair, unconscious, having clonic spasms of the entire body — most marked in the muscles of the back — so severe as to require three men to hold him and prevent his injuring himself and those about him.

I had him removed at once to the bed, and placed in the recumbent posture, when I began administering chloroform by inhalation, which soon quieted and brought him under control.

Upon inquiry found that he had attended a Sabbath School Concert the evening previous, from which he returned feeling perfectly well. Before retiring, he ate quite a hearty meal which seemed to distress him considerably, as he was restless and unable to sleep soundly. At 2 A. M., he arose and went down stairs to procure a drink of water, stating to his companion that he felt sick at his stomach. While down stairs he was attacked with spasms which he continued having for a half hour after my arrival.

From the history ascertained, I concluded that the hearty meal taken just before retiring was the exciting cause; consequently I administered an emetic which I was obliged to repeat before procuring the desired effect. The second dose being effectual, caused him to eject quite a quantity of undigested meat, etc., etc. This seemed to ease him completely, as he soon returned to consciousness, the spasms ceasing, and leaving him perfectly rational.

8 A. M. Found him feeling well, pulse natural, has slept some since my previous visit.

5 P. M. Patient sitting up, slept considerably during the day and is gaining rapidly. Thinking all would go well and that simply a good night's rest was all that was required, I left some Dover's powder to be taken at bed time, providing he did not rest well. To my surprise, I was summoned in haste at 7 P. M., of the same evening. The patient was again in spasms.

I was soon at his side administering chloroform. The spasms were not so severe as those of the morning previous and they succumbed more readily to the influence of chloroform. I commenced giving fifteen grain doses of bromide of potassium every two hours. The pulse during as well as intervening the spasms, was at that time natural. At 9 P. M., he had another spasm; at 11:30 P. M., another, and at 4 A. M., May 9th, still another. I now began giving, alternately with the bromide, seven and one-half grain doses of hydrate of chloral.

He seemed now to rest well, complaining of nothing except a soreness in the muscles from excessive straining. At 12:30 P. M., he had another spasm. I commenced giving two grains of assafœtida every hour, in lieu of the bromide, continuing the chloral.

The following night, *i. e.*, May 9th, he had three of these spells, regularly at the stated hours of the night previous. After the third one, which was at 11:30 P. M., his pulse was slow and full, and he was more drowsy. I now bled him some ℥ viij, and commenced giving quiniæ sulphatis. grs. ij, every hour during the day, alternating with the assafœtida, thinking he might be malarious, as the spasms seemed to assume a periodical type, gave the chloral p. r. n.

No more spasms occurred until 11 P. M., May 10th, at which time they were very light. Pulse slow and full, patient drowsy. I again bled him some two ounces more than upon the previous night, which rapidly improved the symptoms quite perceptibly.

May 11th. Patient feeling quite well, sitting up most of the time. Appetite good, says he cannot eat enough.

Friday, May 12th. Patient up about the house and yard, in the afternoon about town.

Saturday, May 13th. He has left town to take a few days of rest and country air.

